# A non-randomized pilot study to test the feasibility of treating chronic pain and opioid prescription use in rural areas with acceptance and commitment therapy (T-PACT)

**DOI:** 10.1017/cts.2020.26

**Published:** 2020-03-24

**Authors:** Robert L Rhyne, Heidi Rishel Brakey, Jacquie R. Halladay, Kathleen Mottus, K. Allen Greiner, Elizabeth Salt, Orrin Myers, Kent Sutton, Jesus Fuentes, Kevin E. Vowles

**Affiliations:** 1 Department of Family and Community Medicine, Community Engagement and Research Core, Clinical and Translational Science Center, University of New Mexico Health Sciences Center, Albuquerque, NM, USA; 2 Clinical and Translational Science Center, University of New Mexico Health Science Center, Albuquerque, NM, USA; 3 Department of Family Medicine, University of North Carolina School of Medicine, Chapel Hill, NC, USA; 4 North Carolina Translational and Clinical Sciences (NC TraCS) Institute, University of North Carolina at Chapel Hill, Chapel Hill, NC, USA; 5 Department of Family Medicine, Kansas Patients and Providers Engaged in Prevention Research (KPPEPR), University of Kansas Medical Center, Kansas City, KS, USA; 6 College of Nursing, University of Kentucky, Lexington, KY, USA; 7 Department of Family and Community Medicine, University of New Mexico Health Sciences Center, Albuquerque, NM, USA; 8 Cecil G. Sheps Center for Health Services Research, The University of North Carolina at Chapel Hill, Chapel Hill, NC, USA; 9 Centre for Improving Health-Related Quality of Life, School of Psychology, Queen’s University Belfast, Belfast, UK

**Keywords:** Feasibility study, pilot study, opioid, chronic non-cancer pain, acceptance and commitment therapy

## Abstract

Chronic non-cancer pain (CNCP) involves one-third of the US population, and prescription opioids contribute to the opioid epidemic. The Centers for Disease Control and Prevention emphasizes maximizing non-opioid treatment, but many rural populations cannot access alternative therapies. Clinical and Translational Science Award hubs across four rural states performed a multi-site, single-arm intervention feasibility study testing methods and procedures of implementing a behavioral intervention, acceptance and commitment therapy, in primary care CNCP patients on chronic opioids. Using the CONSORT extension for feasibility studies, we describe lessons learned in recruiting/retaining participants, intervention implementation, data measurement, and multi-site procedures. Results inform a future definitive trial and potentially others conducting rural trials.

## Introduction

Chronic non-cancer pain (CNCP) affects approximately one-third of the US population. Opioids have been overprescribed for CNCP resulting in the current “opioid overdose crisis,” as described by the Centers for Disease Control and Prevention (CDC) [1]. Overprescribing became widespread partly as a result of actions by the pharma industry [2], despite strong evidence for nonpharmacological pain treatment, and a lack of evidence that medication treatment alone optimizes function and long-term outcomes for CNCP patients [3,4]. Many rural regions of the country have been especially impacted by the opioid crisis [5].

CDC opioid-prescribing guidelines emphasize maximizing non-opioid therapy before prescribing opioids [6]. However, poor access to healthcare services in rural areas has contributed to limited use of nonpharmacological treatments. Behavioral interventions are underutilized in CNCP management. Acceptance and commitment therapy (ACT) is a type of behavioral counselling where patients learn to focus on their present situation with CNCP, accept their condition, and find positive coping skills [7]. ACT has been effective for chronic pain [8,9] but has not been tested in rural primary care settings.

Some of the same barriers to performing research in rural areas are also associated with health disparities, namely geographic isolation, shortage of healthcare providers, poverty, transportation difficulties, and less access to healthcare [10]. Also, translational research and recruitment for trials in rural clinical settings are limited by a lack of research infrastructure, awareness, education, and experience by providers, as well as competing demands on clinic personnel and workflow [11].

Feasibility studies are crucial for planning effectiveness trials, investigating proposed methods, and determining if trials can be performed [12]. Specific CONSORT-reporting guidelines and checklists have been extended to randomized feasibility studies, and certain principles can also be used for non-randomized pilot studies [13,14]. Feasibility studies allow study teams to gauge potential hurdles in performing larger, multi-site, pragmatic randomized controlled trials (RCTs) [15].

Our research team, comprised of investigators from four Clinical and Translational Science Award (CTSA) hubs, collaborated to perform this non-randomized pilot study in rural areas of North Carolina, Kentucky, Kansas, and New Mexico among primary care patients with CNCP who were treated with prescription opioids. The aim of this study was to evaluate the feasibility of testing ACT as a behavioral intervention and evaluating the multi-site study procedures for a future RCT. Specifically, our objectives for this study focused on the following major CONSORT checklist items: single IRB, recruitment and retention of participants through practices, implementation of the ACT intervention, and collection of study data across sites.

## Methods

The Rural Health Research Support Network [16] is a collaborative effort among CTSA hubs to facilitate and provide support for multi-site CTR studies in rural populations. It led to the collaboration of four CTSA hubs to design and conduct this feasibility study at the Universities of New Mexico (UNM), North Carolina, Kansas, and Kentucky. This study was approved using SMART IRB with UNM as the reviewing IRB [17]. The study was registered with ClinicalTrials.gov (ID: NCT03666455). Each CTSA funded the study at $25,000 ($100,000 total).

This non-randomized pilot study tested the feasibility, methods, and procedures of implementing ACT across four sites. The implementation period for this study was January through September 2019. It is in progress as some data collection is ongoing. Each site recruited one rural primary care practice (PCP) with behavioral health support availability within or closely associated with the practice. At least one master’s level or higher behavioral health therapist per site was recruited and trained to deliver the ACT intervention. Our target sample size was 5 patients per site, or 20 totally, using the following eligibility criteria: age 21–65, diagnosis of CNCP, chronic prescription opioid use, ability to complete an extensive survey at three time points, and ability to attend all ACT therapy sessions. To allow for dropouts, our aim was to recruit seven patients from each PCP in order to retain five through all ACT sessions. Each site team oriented their PCP to the study and its methods, and relied primarily on providers for recruitment by identifying and distributing study flyers to prospective participants, which had a contact number for study personnel.

ACT is an empirically supported [9], structured, eight-session behavioral therapy designed to help patients accept their CNCP and commit to positive coping skills. Co-founder KV trained therapists over eight 2-hour sessions using a treatment manual [7] and videoconferencing platform. The training covered the concepts and theory of each session, its goals and objectives, treatment methods, and application. Sessions were audio-recorded for the purposes of clinical supervision and intervention fidelity assessment.

Patients were to complete baseline surveys including demographics and standardized measures, eight sessions of ACT, and a brief pain measure at each therapy session. Follow-up measures and semi-structured interviews were scheduled to be collected after the intervention but are not included here because they were not part of the intervention period being tested in this study. The standardized baseline measures included pain outcome measures, comorbidity behavioral health measures, and prescription opioid aberrant behavior measures. Pain measures included the Brief Pain Inventory (BPI) and its brief version, the Pain, Enjoyment of life and General Activity (PEG), both of which measure pain intensity and interference with daily function. The BPI was collected at baseline and the PEG was collected at each ACT session. Other measures included the Short Form Health Survey (SF-36), a measure of functional health and well-being; the Patient Health Questionnaire depression scale (PHQ-8); the Chronic Pain Acceptance Questionnaire – Revised, a measure of the acceptance of chronic pain; the Chronic Pain Values Inventory, a measure based on the values-based ACT treatment; the Current Opioid Misuse Measure, used to monitor pain patients on opioids regarding aberrant medication behaviors; and the Brief Adverse Childhood Events measure.

Investigators elicited feedback on study and implementation methods from research staff and PCP teams. At scheduled team videoconferences twice monthly, we discussed these issues and generated a list of key themes, best practices, and lessons learned in performing this multi-site feasibility study.

## Results

Of the targeted number of 20–28 participants, we recruited 21 totally (Table 1). The majority were female, Non-Hispanic White, over 50 years of age, high school or higher educated, and in the $50,000 yearly income range. Of the 21, only four, or 19%, dropped out: two in Kansas after the first therapy session; and two in New Mexico, one after seven sessions and one no show after enrollment.

**Table 1.** Baseline recruitment by study site and participant demographics

Measure	Frequency (*n*_*t*_ = 21): *n* (%)
Sites	
Kansas	7 (33.3)
Kentucky	5 (23.8)
New Mexico	3 (14.3)
North Carolina	6 (28.6)
Participant demographics	
Female	14 (66.7)
Race/Ethnicity	
Non-Hispanic White	20 (95.2)
Black	1 (4.8)
Age, years	57 (42–70)
Education	
Less than high school	1 (4.8)
High school or GED	9 (42.9)
Some college – less than 4-yeardegree	6 (28.6)
College degree	5 (23.8)
Household income	
Less than $25,000	6 (28.6)
$25,000–$49,000	5 (23.8)
$50,000 or more	10 (47.6)

One of the recruited participants was in the early stages of the ACT sessions at the time of this study, so Table 2 shows follow-up data for 20 participants who had the time in study to finish ACT sessions. It includes the four dropouts. In Kansas, the 2 dropouts missed 14 sessions, and in New Mexico (NM), 2 dropouts missed 9 sessions. All others completed their sessions. This resulted in an overall ACT completion rate of 86%. The overall PEG collection the rate was 91%; 5 of the remaining non-dropouts in NM did not complete a PEG during their ACT sessions; 8 in North Carolina.

**Table 2.** ACT intervention and PEG outcome measure frequency of completion

Study site	Number of patients^a^	ACT available sessions	ACT completed sessions	%	PEG collection	%
Kansas	7	56	42	75	42	100
Kentucky	4	32	32	100	32	100
New Mexico	3	24	15	63	10	67
North Carolina	6	48	48	100	40	88
Total	20	160	137	86	124	91

a One recruited Kentucky patient excluded because of insufficient time available to complete therapy sessions.

Table 3 shows the CONSORT extension checklist items evaluated in this study.

**Table 3.** Challenges, lessons learned, and solutions by CONSORT checklist item

Study issue	Challenges	Solutions and lessons
IRB		
Single IRB (sIRB)	New infrastructure and processes to initiate	Modifications easier and better with team approach
Recruitment and retention of patients		
Eligibility criteria	Patient reluctance to decrease opioids	Remove as goal in materials, consider in therapy
	Age ≤ 65 years	Expand to >18 years
	Illicit drugs not included	Future, different study for this population
Outreach	Referrals from providers (competing demands)	Clinic staff for recruitment, extend beyond primary care, recruitment outside clinic setting (e.g., radio ads, printed material)
Retention	Transportation in rural areas	Telemedicine, reimburse travel costs
Practice level	Clinic not prioritizing study	Sustained engagement
Recruitment	Stigma with “counselling” language	New terms to describe, therapist part of PCP structure
Intervention implementation: training/support for ACT therapists		
Training therapists	Train therapists across states	Videoconferencing worked well
	Training before enrollment	Align with patient sessions, form training network
	Therapists not in clinics	Use community resources, i.e., school counselors
Protecting therapist time	Paying for time did not work out as planned	Dividing effort among team of therapists, better communication with management
	Regulations for reimbursement	Do not allow clinics to charge regular fees and co-pays, use study funds to reimburse therapist time
Intervention implementation		
Scheduling	Issue for rural patients	Flexibility in timing/number of visits (16 vs 12 weeks)
Locations	Only used primary care clinic sites	Consider telemedicine ACT sessions
Survey	Sensitive questions asked	Need respectful way to deal with; are questions about illicit opioid misuse needed?
Baseline measurement/data collection		
Data collection	Distance from university made data collection difficult	Train clinic staff, therapists in research methods, data collection
	Need centralized way to collect data across states	REDCap worked, but data access and sharing was difficult with university firewall
	Time for survey due to tech and health literacy	Allow patients to complete over phone, may need to simplify language, and reduce number of measures
	Therapy audio files for fidelity	Used REDCap, but issues with file size; permissions had to be changed for audio files
Study administration		
Coordinating researchers	Staff across states	Used regular videoconferences
Coordinating clinics	Communication between researchers and clinics	Institute a regular huddle, include in-person visits

### Single IRB

This study was the first project at UNM to use single IRB as a prime site [17]. The process to obtain approval was challenging, but close communication between the research teams and their IRBs facilitated a successful process. Subsequent modifications were centralized, which facilitated a better process.

### Recruitment and Retention

The recruitment flyer stated the goal of decreasing opioid use, something we quickly learned, was a deterrent for those taking chronic prescription opioids. Initially, the upper age limit was 65 years, but we learned there were many older eligible patients and changed inclusion to anyone over the age of 18. We relied on PCP providers for patient referrals, but because of competing demands, this resulted in very slow recruitment, and they suggested we use other clinic staff to help recruit. We did expand recruitment to the therapists who helped increase enrollment.

### Intervention Implementation: Training/Support for ACT Therapists

Remote ACT training was challenging, given the amount of time required and topic sensitivity. Videoconference sessions were effective, with positive feedback from therapists. Since the training concluded before any ACT sessions were conducted, therapists suggested relevant session-specific reinforcement occurs prior to respective patient sessions.

Rural areas are often challenged by having limited resources. One clinic chose to use health insurance to fund therapist time, rather than accepting study reimbursement. Upon being charged a co-pay, one patient withdrew. After 6 months of negotiation and delayed recruitment, the clinic agreed to revert to study reimbursement.

### Intervention Implementation

Some patients had difficulty with transportation and scheduling. Despite this, and dropouts, we had an 86% completion rate (Table 2). Flexibility in the timing of sessions and expanding the time to complete all sessions from 12 to 16 weeks helped retain participants. Some measures activated sensitive issues in patients, i.e., adverse childhood experiences. Expanding therapist training and support sessions during the process of therapy will address this in future studies. Since some therapists did not have availability of an acute mental health unit to refer a suicidal patient, we used the validated PHQ-8 [18] instead of the PHQ-9, which includes a suicidality question.

### Baseline Measurement/Data collection

Our multi-site data collection system used Research Electronic Data Capture (REDCap) [19], a browser-based metadata-driven software, to collect and transmit data to a centralized secure server, where an analyzable database was built. To avoid the onerous process of non-UNM study team members obtaining UNM logins and VPN access, data were submitted through a survey link. Therefore, UNM owned the REDCap data, but de-identified site-specific data can be shared.

Collecting numerous surveys remotely by phone proved time consuming and difficult. In-person or phone surveys were administered by research staff when patients had difficulty completing surveys due to health literacy or lack of technology.

### Study Administration

Team building fostered trust and engagement, which is important working with a large team across multiple sites and rural practices. We conducted twice monthly videoconferences among site research teams.

## Discussion

Lessons learned from this feasibility study provide valuable information to inform a larger, definitive pragmatic trial on ACT as an alternative treatment for rural CNCP patients taking chronic opioids. Recruitment was difficult. Some patients feared joining our study with the stated goal of decreasing opioids. Because the principal aim of a future study will be on testing an alternative treatment for coping with CNCP, not primarily on opioid reduction, we will not mention opioid reduction in future recruitment materials. Instead, because it will remain a secondary goal, we will make it a recruitment criteria and consideration during the behavioral intervention.

Retention showed good feasibility, 86% for completing ACT therapy sessions and 91% for completing PEG pain measures. However, as expected in rural areas, transportation and scheduling visits outside usual primary care were problematic. Possible solutions include conducting telemedicine therapy sessions after at least one in-person visit and reimbursing transportation costs. Some rural participants do not have electronic connectivity, and some had trouble completing the surveys because of health literacy. To improve outcome measurement, we will collect more data in person or by phone when needed, ask questions in more accessible language, and collect the data using more than one session.

Supporting therapists who deliver this intervention presented unexpected barriers. Some patients recruited for “counselling” felt stigmatized, feeling they had a mental health problem. Improved messaging can reduce misconceptions. Training by videoconference worked well, but individualized support during therapy and establishing a training network would help improve the delivery of the intervention. Protecting therapist time and scheduling is a priority because of patient load and competing demands in rural areas. We will consider splitting research funding among multiple therapists so they have a built-in team, can cover one another, and potentially have greater influence on practice leadership.

Study administration in rural multi-site PCPs requires a high level of engagement between researchers and practices [20]. Research teams should have ongoing communications with practice leadership, providers, and staff regarding study objectives and progress. In performing practice-based research, compensating practices for their time, resources, space, and interference with workflow helps with practice engagement.
